# Inhibitory Effects of *Tenebrio molitor* Larva Trypsin Hydrolysate on Androgenetic Alopecia

**DOI:** 10.4014/jmb.2606.06002

**Published:** 2026-08-14

**Authors:** Seok-Hee Kim, Young-Jin Choi, Nishala Erandi Wedamulla, Qun Zhang, Jin-Hwan Lee, Eun-Kyung Kim

**Affiliations:** 1 Department of Applied Bioscience, Graduate School of Dong-A University, Busan 49315, Republic of Korea; 2 Jeju Institute of Korean Medicine, Jeju 63309, Republic of Korea; 3 Department of Food Science and Technology, Faculty of Animal Science and Export Agriculture, Uva Wellassa University, Badulla 90000, Sri Lanka; 4 Division of Food Bioscience, Konkuk University, Chungju 27478, Republic of Korea; 5 Department of Smart Green Resources, Dong-A University, 37, Nakdong-Daero 550 Beon-gil, Saha-gu, Busan 49315, Republic of Korea; 6 Nutritional Education Major, Graduate School of Education, Dong-A University, Busan 49315, Republic of Korea; 7 Nutrinomics Lab. Co. Ltd., Busan 49315, Republic of Korea; 8 Center for Food & Bio Innovation, DAU G-LAMP Project Group, Dong-A University, Busan 49315, Republic of Korea

**Keywords:** Androgenetic alopecia, *Tenebrio molitor*, Wnt/β-catenin signaling, Hair loss, Cell proliferation

## Abstract

Androgenetic alopecia (AGA) is the most common form of hair loss, with a high prevalence worldwide. In this study, we aimed to investigate the potential of *Tenebrio molitor* larva trypsin hydrolysate (TMTH), an insect-derived bioactive hydrolysate, as a functional ingredient candidate for AGA. *In vitro* and *in vivo* assessments demonstrated that TMTH promoted human hair dermal papilla (HDP) cell proliferation, restored β-catenin expression, and alleviated dihydrotestosterone (DHT)-induced inhibition. In a testosterone propionate (TP)-induced alopecia mouse model, oral TMTH administration increased hair coverage and follicle number, upregulated Wnt/β-catenin and PI3K/Akt signaling, reduced androgen receptor expression, enhanced antioxidant-related responses, and suppressed inflammatory markers. These findings suggest that TMTH may ameliorate AGA-related phenotypes by modulating hair growth-related signaling, local androgen-related responses, oxidative stress, and inflammation, thereby supporting its potential as a functional ingredient candidate for hair loss management.

## Introduction

Alopecia is a long-term skin condition that leads to partial or complete hair loss on the scalp and, in some cases, other areas of the body. It is also identified as a chronic inflammatory disease that targets hair follicles [1]. Overall, the primary types of alopecia are alopecia areata, telogen alopecia, and androgenetic alopecia (AGA), with the latter being the most common [2]. The mechanism of this disease is linked to the response to androgens. The distribution of androgen receptors in the scalp affects the distinctive pattern of hair loss in both men and women [3].

Generally, hair loss and replacement are a cycle, where hair follicles progress through corresponding phases of growth, involution, quiescence, and regeneration. The growth phase is called the anagen phase that lasts for 3 to 5 years, while the involutional phase, named catagen comes at the end of the anagen phase and lasts for a few weeks. The telogen phase follows the catagen phase and lasts for approximately 3 months. Hair follicle quiescence occurs during this period. Hair follicle regeneration occurs during the initial week of the anagen phase, and once this is complete, the anagen phase proceeds until the hair reaches its final length [4]. A decrease in the duration of the anagen phase results in shorter hair length, whereas an extended telogen phase postpones regeneration. This results in hairs that are so short and fine that they cannot grow long enough to reach the surface of the scalp, leading to a greater number of empty follicles [5].

The hair growth cycle is regulated by a variety of molecular signals, such as growth factors, nuclear receptors, cytokines, and intracellular signaling pathways. Among these, activation of the Wnt/β-catenin signaling pathway upregulates the expression of downstream target genes, including hepatocyte growth factor (HGF), vascular endothelial growth factor (VEGF), and insulin-like growth factor-1 (IGF-1), thereby inducing the anagen phase in dermal papilla cells and promoting hair follicle regeneration [6-8]. In contrast, transition to the catagen phase has been associated with a decline in anagen-maintaining factors, such as IGF-1, basic fibroblast growth factor (bFGF), and VEGF, along with an increased expression of apoptotic cytokines, including tumor necrosis factor alpha (TNF-α) [9-10]. Although AGA is common and significantly affects patients, currently available approved treatments, such as finasteride and minoxidil, have limitations [11-12]. Finasteride is associated with adverse effects, including erectile dysfunction, decreased libido, and ejaculatory disorders, which may pose concerns regarding long-term compliance. Minoxidil has been reported to cause scalp irritation, itching, and unwanted facial hair growth, particularly in susceptible individuals [13]. Therefore, alternative treatment options with improved tolerability are needed, and natural functional ingredients have gained attention as potential candidates for alopecia management.

Edible insects provide a wide range of nutrients, including protein, lipids, essential minerals, vitamins, and fiber, with proteins and fats as the main nutritional components [14-15]. Recently, edible insects have been identified as promising sources of bioactive compounds owing to their unique nutritional composition [16]. Protein hydrolysates are peptide-rich mixtures generated by enzymatic digestion of proteins. Enzymatic hydrolysis can improve solubility, digestibility, absorption, and biological activity by producing low-molecular-weight bioactive peptides. *T. molitor* larva-derived peptides have demonstrated diverse bioactive properties, such as anti-obesity [17], anti-inflammatory [18], antioxidant [19], and antihypertensive [20] effects. They can potentially prevent muscle atrophy as well [21]. Nevertheless, their effectiveness in treating AGA has not been fully explored. In the current study, we aimed to investigate the inhibitory potential of *T. molitor* larva trypsin hydrolysate (TMTH) on AGA using human hair dermal papilla (HDP) cell proliferation and wound healing assays. TMTH was further evaluated for its restorative effects under DHT-treated conditions. *In vivo* experiments were conducted using C57BL/6 mice with testosterone-induced hair loss, and TMTH was orally administered to evaluate its inhibitory effect on AGA-related phenotypes.

## Materials and Methods

### Materials

DMEM and fetal bovine serum (FBS) were purchased from Welgene (Republic of Korea). Penicillin/streptomycin (P/S) was obtained from Gibco (USA). Testosterone propionate (TP) was provided by the Tokyo Chemical Industry Co. (Japan). Dihydrotestosterone (DHT) was purchased from Sigma-Aldrich (USA). Minoxidil (MNX) was purchased from Daejung (Republic of Korea). β-Actin, AR, Akt, p-Akt, Wnt3a, PI3K, Nrf2, HO-1, TNF-α, and β-catenin antibodies were purchased from Santa Cruz Biotechnology (USA). Antibodies against p-GSK3β and GSK3β were purchased from Cell Signaling Technology (USA). Horseradish peroxidase-conjugated goat anti-rabbit IgG, horseradish peroxidase-conjugated goat anti-mouse IgG, and FSD™ 594 conjugated goat anti-rabbit IgG were acquired from BioActs (Republic of Korea).

### Preparation of Enzymatic Hydrolysates of Edible Insects

The hydrolysates used in this study were supplied by Gyeongnam Agricultural Research and Extension Services, Jinju, Republic of Korea. The enzymatic treatment of substrates was performed using a standardized protocol. A 10% (w/v) suspension of the substrate (freeze-dried and ground *T. molitor* larvae) was prepared by mixing 10 g of substrate with 100 mL of distilled water (DW), followed by treatment with 1% (w/w) enzyme, corresponding to 100 mg of enzyme per 10 g of substrate. The suspension was incubated at 38°C for trypsin (TRY) or at 50°C for FoodPro alkaline protease (AK) and Protease NP (PNP). Enzymatic activity was terminated by heating the reaction mixtures in a water bath at 80°C for 10 min.

### Cell Culture

HDP cells were obtained from Cell Engineering for Origin (Republic of Korea). The cells were cultured in DMEM containing 10% FBS and 1% P/S in a humidified atmosphere of 5% CO_2_ at 37°C. HDP cells were seeded into 6-well plates (5 × 10^5^ cells/well) in 2 mL of DMEM containing 10% FBS and 1% P/S. After 24 h, the cells were co-treated with DHT (10 μM) and TMTH (12.5, 25, or 50 μg/mL) for 24 h. HDP cells treated with MNX (50 μM) were used as positive controls. The cells were finally collected for immunofluorescence, wound healing assay, and western blot analysis.

### Cell Viability

HDP cells were seeded into 96-well plates at a density of 5 × 10 cells per well and incubated at 37°C in a 5% CO_2_ atmosphere. After 24 h of incubation, the cells were treated with DHT (10 μM) and MNX (50 μM), followed by different concentrations of TMTH (12.5–2,000 μg/mL) for 24 h at 37°C. After treatment, MTT solution (5 mg/mL) was added to each well and incubated for 4 h. The medium was then removed, and dimethyl sulfoxide (100 μL) was added to dissolve the formazan crystals. Absorbance was measured at 540 nm, and cell viability was calculated as a percentage relative to untreated wells.

### Wound Healing Assay

HDP cells were seeded into 6-well plates at a density of 5 × 10^5^ cells/well and incubated in a humidified atmosphere of 5% CO_2_ at 37°C for 24 h. Wounds were induced on the monolayer with a 1,000-μL pipette tip. HDP cells were photographed using a Leica microscope (Germany), and the healed areas were estimated using ImageJ (National Institutes of Health, USA) after treatment with DHT (10 μM), TMTH (12.5, 25, or 50 μg/mL), and MNX (50 μM) for 24 h.

### Immunofluorescence

HDP cells were treated with TMTH (12.5, 25 or 50 μg/mL). β-catenin expression was visualized using immunofluorescence. Briefly, HDP cells (5 × 10^3^ cells/well) were seeded into an 8-well slide chamber (SPL Life Science Co., Republic of Korea). The cells were fixed in ice-cold methanol, followed by permeabilization with 0.1% Triton X-100 for 15 min. After blocking in 5% normal goat serum, the slides were incubated overnight at 4°C with β-catenin antibody (1:300 dilution), followed by incubation with the corresponding goat anti-rabbit IgG, FSD 594 (1:1000 dilution) for 1 h. Mounting medium containing DAPI was added to the slides, and coverslips were mounted. Images were captured using a Zeiss 700 confocal microscope (Germany).

### *In Vivo* Experiment Design

Male C57BL/6 mice (n = 40, 6 weeks old) were obtained from Nara Biotech Co., Ltd. (Republic of Korea). The cage setup was carefully controlled under the following conditions: a temperature of 23–25°C, relative humidity of 40–60%, and a light-dark cycle of 12 h, with a 2-h cycle of 40–60% alternating day and night. Water and feed were provided ad libitum. All experimental and animal care procedures were approved by the Dong-A University Animal Care and Use Committee (DIACUC-24-41). To remove back skin hair after a one-week adaptation period, the mice were anesthetized with avertin (50 mg/kg), and the back skin hair was shaved with a clipper and denuded with depilatory cream (Niclean, Ildong Pharmaceutical Co., Ltd., Republic of Korea). After 1 day, AGA was induced by subcutaneous injection (s.c.) of TP (1 mg/kg) dissolved in corn oil daily for 30 days. When TP was administered, TMTH_L (10 mg/kg) and TMTH_H (100 mg/kg) were orally administered daily to the TP+TMTH_L and TP+TMTH_H groups for 30 days. In the TP+MNX group, 5% MNX (5 mg/kg/day) was orally administered for 30 days. After the final injection, the mice were anesthetized with avertin (50 mg/kg) via intraperitoneal injection (i.p.). Sections of the back skin tissue were fixed with 10% formaldehyde for hematoxylin and eosin (H&E) staining, and the remaining back skin tissue was stored at -80°C for western blot analysis.

### H&E Staining

For H&E staining, six back skin tissues were randomly selected from the group of eight. The back skin tissues were fixed with 10% formaldehyde and embedded in paraffin. Sections were cut to a thickness of 5 μm using a Leica microtome (RM 2135, Germany). They were deparaffinized and hydrated prior to staining with hematoxylin for 3 min, and then washed with water for 10 min. The sections were stained with eosin for 1 min. The dehydrated sections were cleared with xylene and mounted. Histological examination was performed using a Leica DMi1 Research Inverted Phase microscope. In each sample, we analyzed three areas under magnification (50).

### Western Blotting

**Fig. 1:**
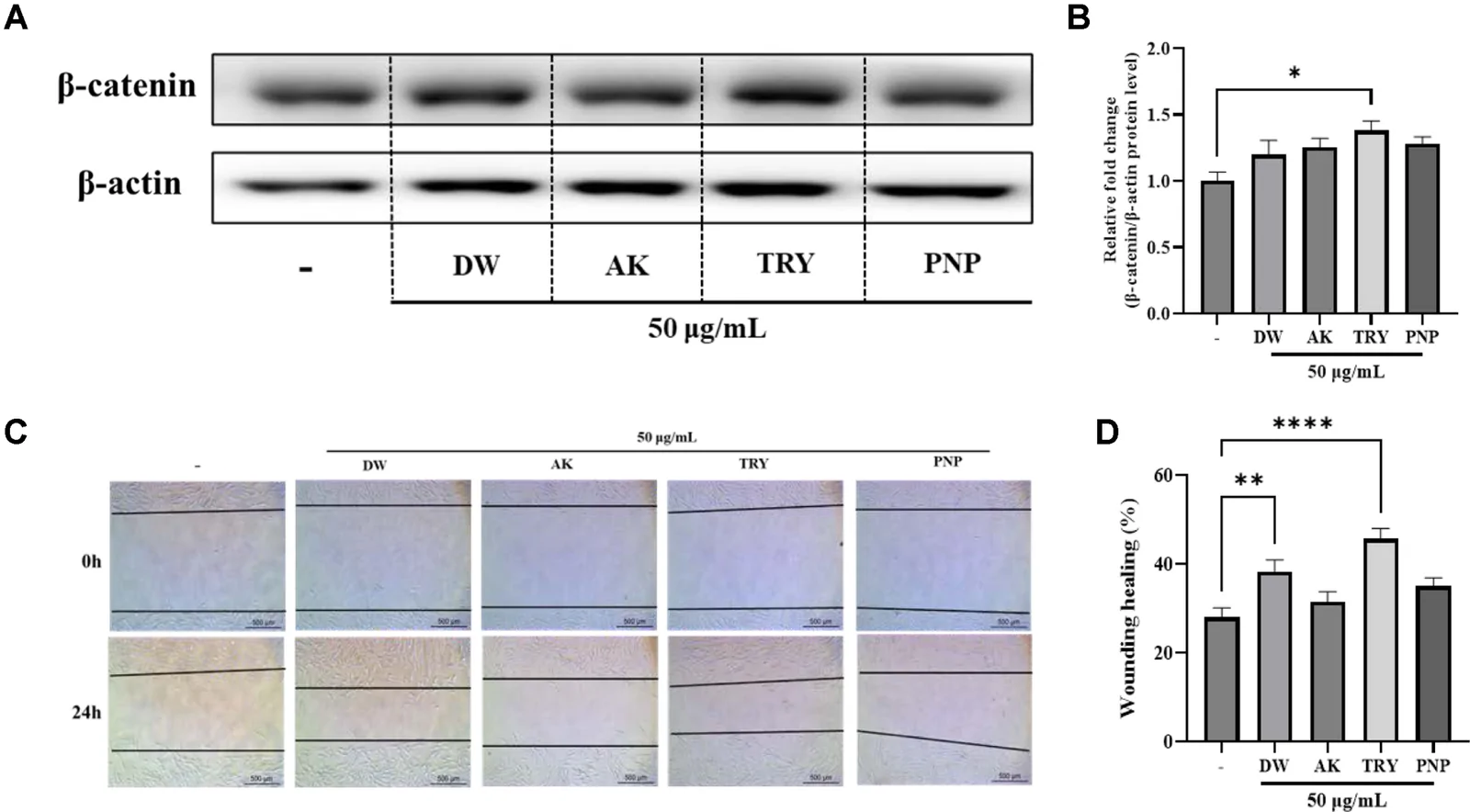
*T. molitor* protein hydrolysates promote β-catenin expression and HDP cell migration. (**A**) Representative western blot images showing β-catenin and β-actin expression in HDP cells. (**B**) Quantitative analysis of β-catenin/β-actin protein levels. (**C**) Representative wound healing images of HDP cells treated with different *T. molitor* hydrolysates. (**D**) Quantitative analysis of wound healing obtained using ImageJ. The membranes were trimmed to match the molecular weight of each target protein, allowing the target proteins and β-actin to be detected on the same membrane. β-actin served as a loading control. Quantitative data are presented from three independent experiments and are expressed as means ± SEM. Statistical analysis was performed using one-way ANOVA followed by Dunnett's test; *p* < 0.05 was considered statistically significant. DW: *Tenebrio molitor* hydrolyzed using heated distilled water, AK: *Tenebrio molitor* hydrolyzed using FoodPro alkaline protease, TRY: *Tenebrio molitor* hydrolyzed using trypsin, PNP: *Tenebrio molitor* hydrolyzed using Protease NP.

HDP cells were harvested and homogenized. Six back skin tissues were randomly selected from a group of eight and lysed with RIPA lysis buffer. The lysates were centrifuged at 13,000 rpm (15,928 g) for 20 min at 4°C, and the supernatants were collected. Bicinchoninic acid assays were used to estimate protein concentrations, and proteins were separated using 10% SDS-PAGE (90 min at 120 V). Proteins were transferred onto nitrocellulose membranes using a Mini Trans-Blot cell (Bio-Rad, USA). After transfer, the membranes were cut at specific molecular weight markers to separate regions containing each target protein. Each cut membrane was blocked with 5% skimmed milk in Tris-buffered saline containing 0.05% Tween 20 (TBST) for 1 h and 30 min at room temperature (25°C). The membranes were incubated with primary antibodies overnight at 4°C. Following three washes with TBST, horseradish peroxidase-conjugated AffiniPure IgG was added and incubated for 1 h and 30 min, followed by three additional washes with TBST for 5 min each. Chemiluminescent signals were detected using a horseradish peroxidase substrate (Advansta, Inc., USA) and an Azure C300 imaging system (Azure Biosystems, USA). Representative western blot images from repeated experiments are presented in the results (Figs. 1, 6, and 7). ImageJ 1.47v was used to quantify chemiluminescent signals. The quantitative histograms present the fold change of each target protein normalized to the corresponding loading or total protein control.

### Scanning Electron Microscopy (SEM)

Field-emission scanning electron microscopy (SCIOS 2; Thermo Fisher Scientific, USA) was used to observe the diameter of dorsal hairs in mice at an accelerating voltage of 5 kV and a working distance of 8 mm. Each sample was coated with gold (10 mA for 120?s) under vacuum using a sputter coater (Cressington 108 Auto Sputter Coater; Cressington Scientific Instruments). All samples were imaged at a magnification of 100.

### Statistical Analysis

Three or more independent experiments are summarized in each figure. Statistical evaluations are expressed as means SEM. The data were analyzed by one-way ANOVA with Dunnett's test using GraphPad Prism (Version 8.4; GraphPad Software, Inc., USA); *p* < 0.05 was considered statistically significant.

## Results

### Effects of Various *T. molitor* Protein Hydrolysates on HDP Cell Migration and β-Catenin Expression

The proliferation of hair dermal papilla cells and the Wnt/β-catenin signaling pathway are crucial for hair growth [22]. Therefore, the proliferation capacity of dermal papilla cells and the expression of β-catenin, a key protein in the Wnt/β-catenin signaling pathway, were examined using a wound healing assay. To evaluate the proliferation effects of various *T. molitor* enzymatic hydrolysates on HDP cells, a wound healing assay was performed. Among the various hydrolysates, TRY hydrolysate showed the highest effect on proliferation, as confirmed by the wound healing assay. Additionally, western blot analysis revealed that the TRY hydrolysate exhibited the highest expression of β-catenin (Fig. 1). Therefore, TMTH was selected for subsequent studies.

### TMTH Stimulates HDP Cell Proliferation and Migration

**Fig. 2:**
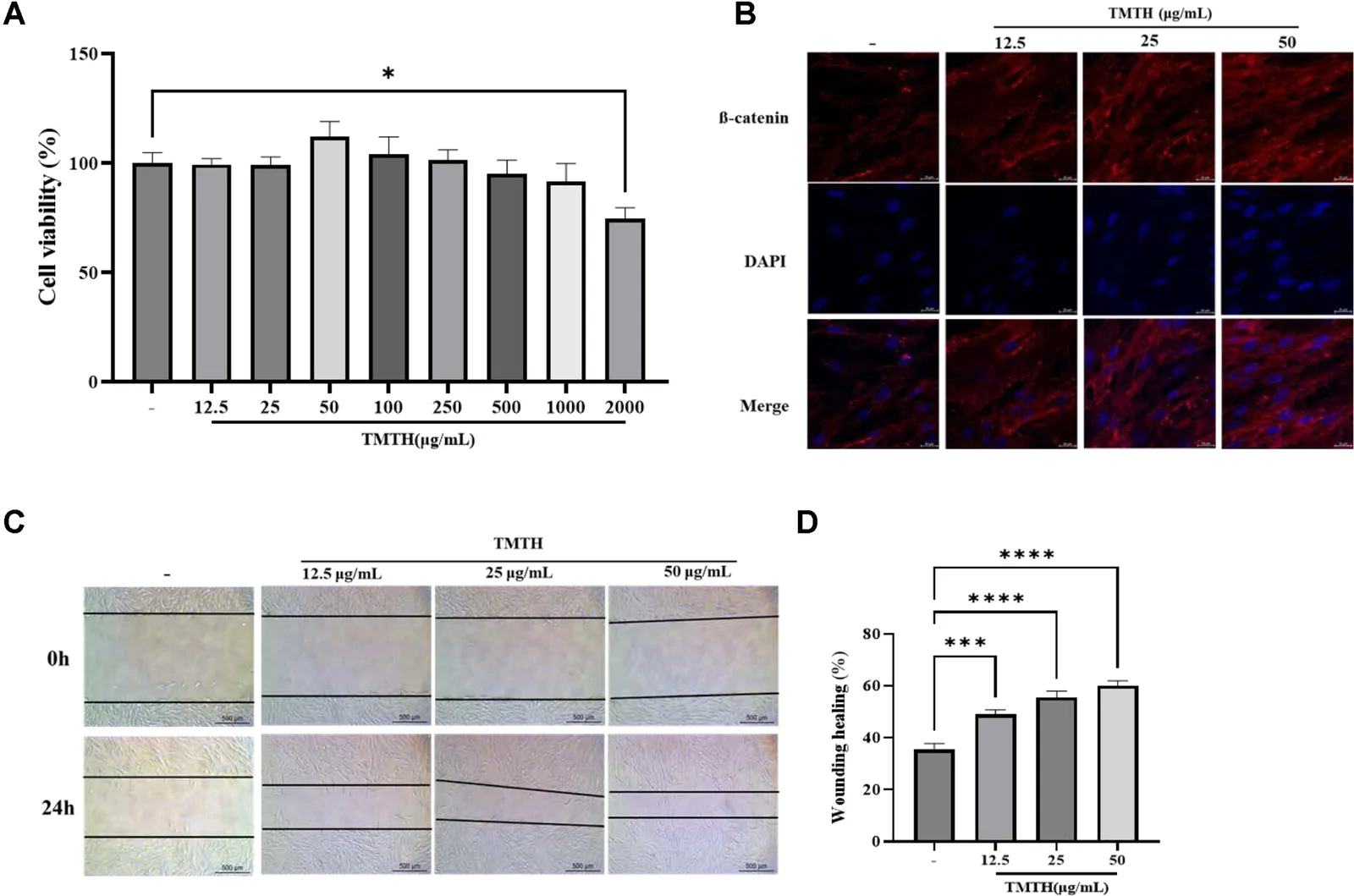
Effects of TMTH on HDP cell proliferation, migration, and β-catenin expression. (**A**) Cell viability of HDP cells after treatment with TMTH for 24 h. (**B**) Representative immunofluorescence images showing β-catenin expression in HDP cells. (**C**) Representative wound healing images of HDP cells treated with TMTH. (**D**) Quantitative analysis of wound healing obtained using ImageJ. Quantitative data are presented from three independent experiments and are expressed as means SEM. Statistical analysis was performed using one-way ANOVA followed by Dunnett's test; *p* < 0.05 was considered statistically significant. TMTH: *Tenebrio molitor* larva trypsin hydrolysate.

To determine the effective concentration range of TMTH, HDP cells were treated with TMTH at 12.5, 25, and 50 μg/mL. Using wound healing and MTT assays, the study confirmed that TMTH promoted HDP cell proliferation and migration in a concentration-dependent manner. Additionally, immunofluorescence staining revealed a concentration-dependent increase in intracellular β-catenin expression (Fig. 2). These results indicate that TMTH increases the expression of β-catenin, a key component of the Wnt/β-catenin signaling pathway, thereby promoting HDP cell proliferation and migration.

### TMTH Restores DHT-Induced Suppression of HDP Cell Proliferation and Migration

**Fig. 3:**
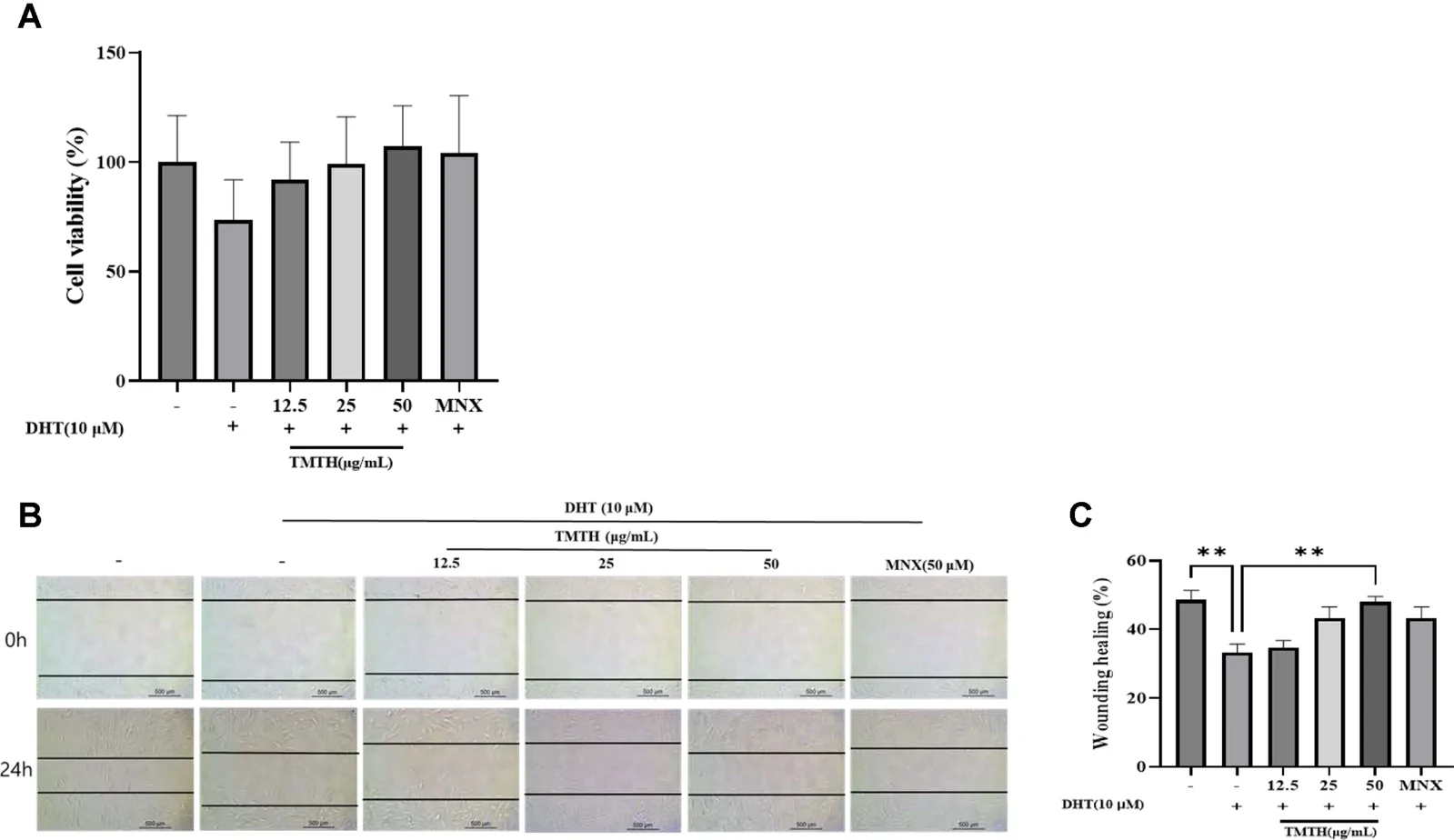
Effects of TMTH on DHT-induced suppression of HDP cell proliferation and migration. (**A**) Cell viability of HDP cells treated with DHT, TMTH, and minoxidil. (**B**) Representative wound healing images of HDP cells. (**C**) Quantitative analysis of wound healing obtained using ImageJ. Quantitative data are presented from three independent experiments and are expressed as means SEM. Statistical analysis was performed using one-way ANOVA followed by Dunnett's test; *p* < 0.05 was considered statistically significant. TMTH: *Tenebrio molitor* larva trypsin hydrolysate, MNX: minoxidil.

TMTH exhibited a hair growth-promoting effect. TMTH was evaluated for its potential to inhibit AGA by enhancing cell proliferation in DHT-induced HDP cells. The wound healing assay revealed a significant reduction in cell proliferation in the DHT-treated group compared with the control group. In contrast, co-treatment with DHT and TMTH resulted in a dose-dependent enhancement of cell proliferation, comparable to or higher than that of the positive control (minoxidil) group (Fig. 3).

### TMTH Ameliorates Hair Growth Inhibition in Testosterone Propionate-Induced C57BL/6 Mice

**Fig. 4:**
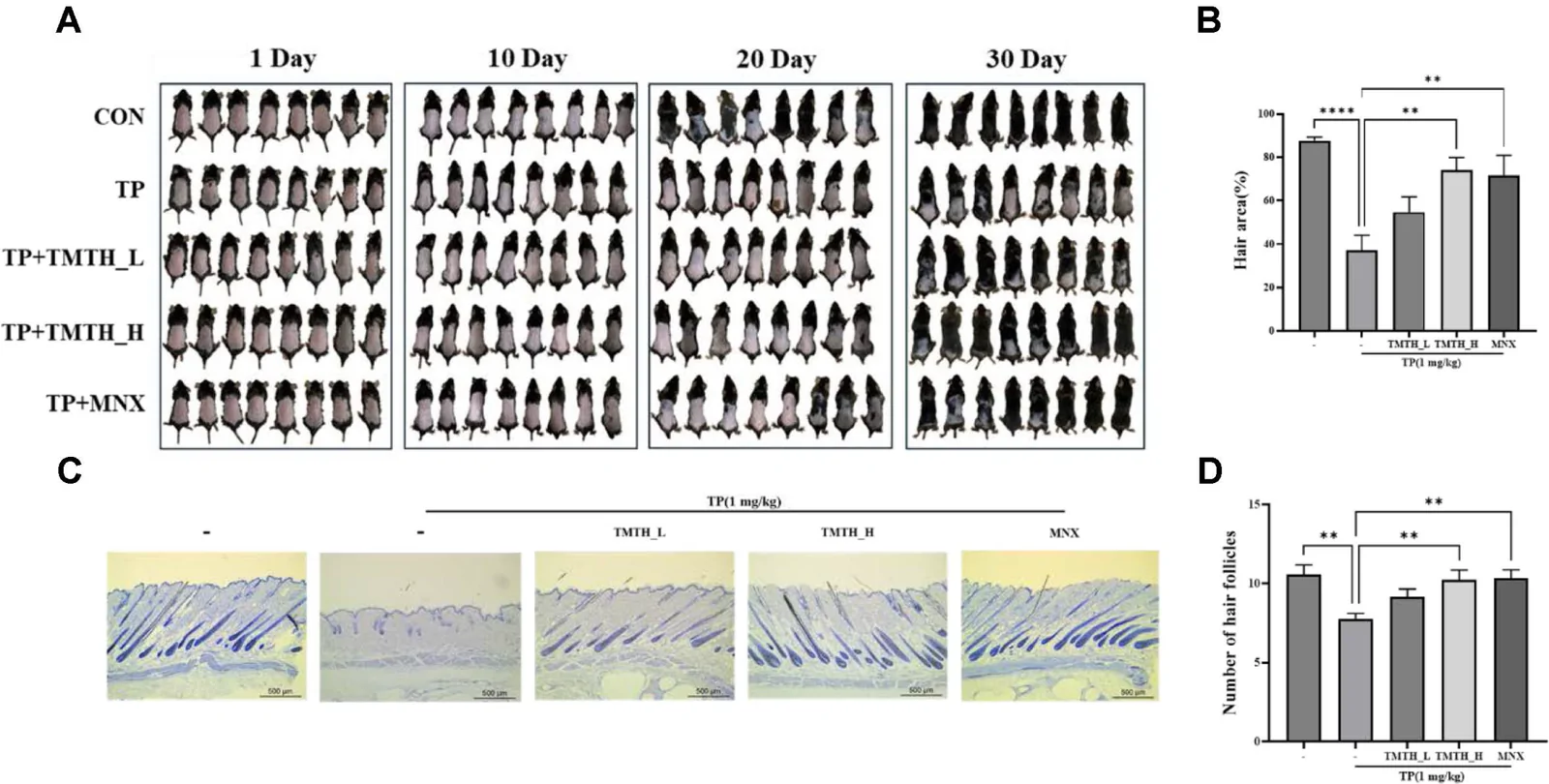
Effects of TMTH on hair growth inhibition in testosterone propionate-induced C57BL/6 mice. (**A**) Images of the dorsal skin were captured on days 10, 20, and 30 following depilation. (**B**) The back skin images on day 30 were analyzed using ImageJ to quantify the regenerated hair area. (**C**) Back skin tissue sections from mice were prepared and analyzed histologically with H&E staining. (**D**) H&E-stained images on day 30 were analyzed using ImageJ to quantify the regenerated number of hair follicles. Quantitative data represent the results obtained from all mice and are presented as means SEM. Statistical analysis was performed using one-way ANOVA followed by Dunnett's test; *p* < 0.05 was considered statistically significant. TMTH: *Tenebrio molitor* larva trypsin hydrolysate, MNX: minoxidil.

The effects of oral TMTH administration on hair growth were assessed in C57BL/6 mice with AGA induced by subcutaneous testosterone injection, as described in previous testosterone-induced hair loss models [23]. After 30 days of oral administration, the control group exhibited complete hair growth. In the TP injection group, hair growth was significantly reduced, confirming successful induction of AGA. In the TMTH-treated groups (10 mg/kg and 100 mg/kg), hair growth was significantly restored compared with the TP group, with the 100 mg/kg dose showing similar hair growth efficacy to that of the positive control group treated with minoxidil. Additionally, H&E staining of dorsal skin revealed that the TMTH-treated groups (10 mg/kg and 100 mg/kg) exhibited larger follicle sizes and a greater number of anagen hair follicles compared with the TP injection group (Fig. 4).

### TMTH Improves Dorsal Hair Thickness in Testosterone Propionate-Induced C57BL/6 Mice

**Fig. 5:**
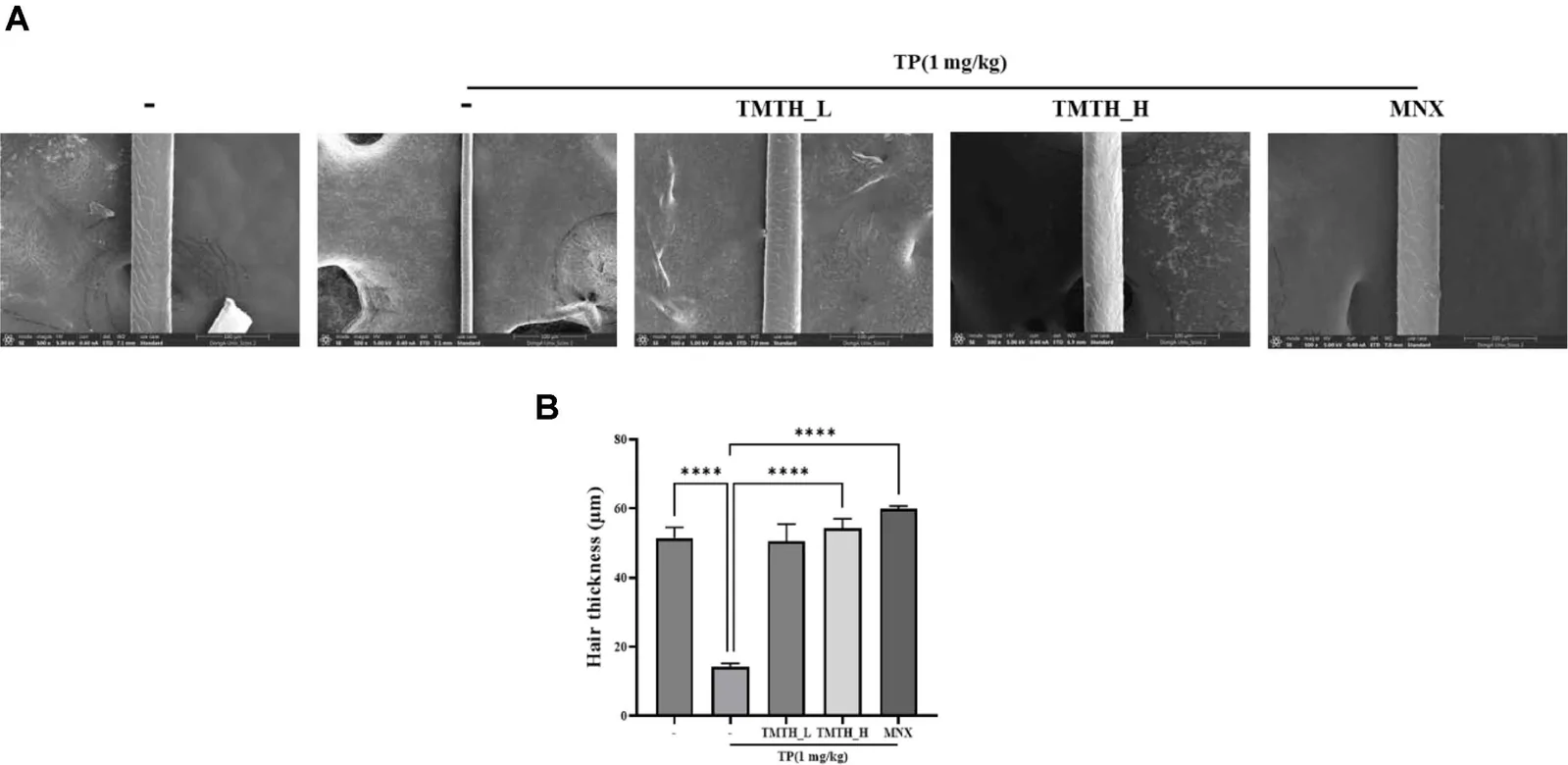
Effects of TMTH on dorsal hair thickness in testosterone propionate-induced C57BL/6 mice. (**A**) SEM images were obtained from mouse dorsal skin hair. (**B**) SEM images were analyzed using ImageJ to quantify hair shaft thickness. Quantitative data represent the results obtained from all mice and are presented as means SEM. Statistical analysis was performed using one-way ANOVA followed by Dunnett's test; *p* < 0.05 was considered statistically significant. TMTH: *Tenebrio molitor* larva trypsin hydrolysate, MNX: minoxidil.

To investigate the impact of TMTH on hair shaft diameter, a C57BL/6 mouse model of AGA was established via the subcutaneous injection of testosterone propionate (TP). After 30 days of oral administration, the control group exhibited normal hair shaft diameter, whereas the TP-injected group showed a significant reduction in hair diameter. In the TMTH-treated groups (10 mg/kg and 100 mg/kg), hair shaft diameter was significantly increased compared with the TP group (Fig. 5).

### TMTH Promotes Wnt/β-Catenin and PI3K/Akt Signaling and Suppresses Androgen Receptor Expression in the Dorsal Skin of Testosterone Propionate-Induced C57BL/6 Mice

**Fig. 6:**
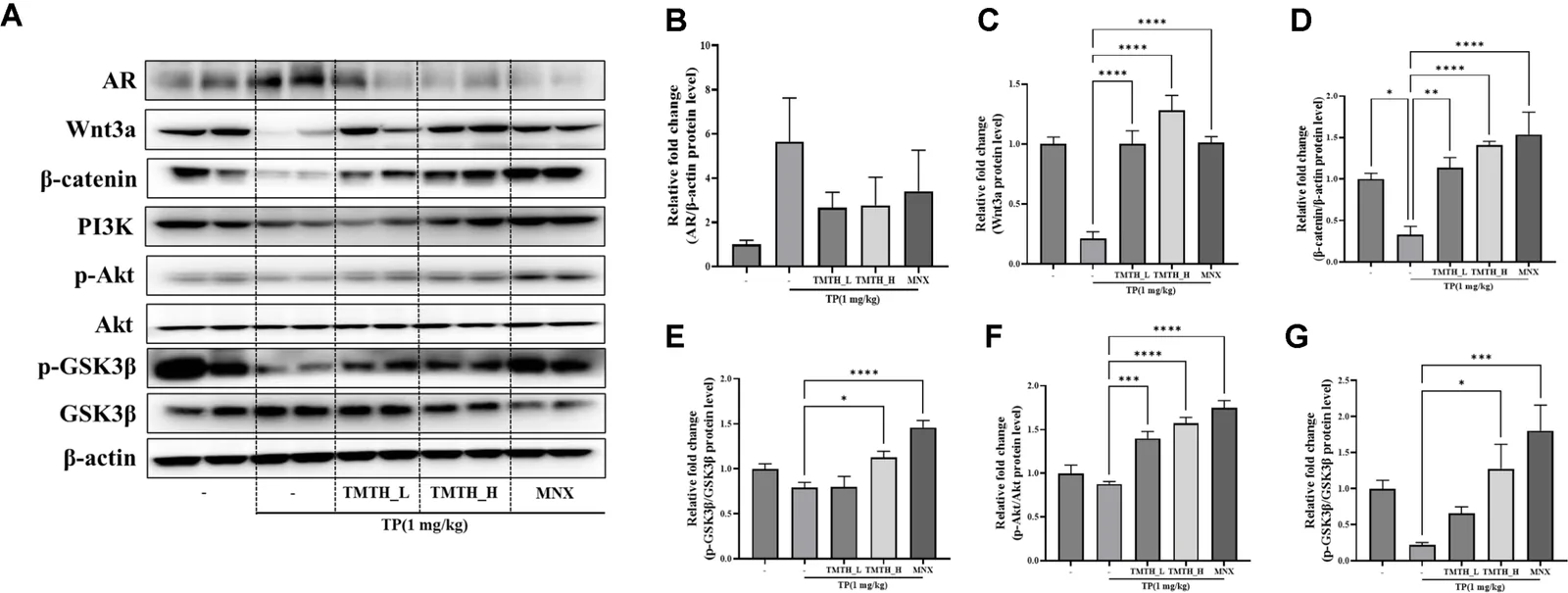
Effects of TMTH on Wnt/β-catenin and PI3K/Akt signaling and AR expression in dorsal skin of TP-induced mice. (**A**) Representative western blot images showing the expression of AR, Wnt3a, β-catenin, PI3K, p-Akt, Akt, p-GSK3β, GSK3β, and β-actin in the dorsal skin of mice. (**B-G**) Quantitative analysis of western blot bands, including AR/β-actin, Wnt3a/β-actin, β-catenin/β-actin, PI3K/β-actin, p-Akt/Akt, and p-GSK3β/GSK3β protein levels. The membranes were trimmed according to the molecular weight of each target protein, allowing target proteins and β-actin to be detected on the same membrane. β-actin served as a loading control. Quantitative data are presented from three independent experiments and are expressed as means ± SEM. Statistical analysis was performed using one-way ANOVA followed by Dunnett's test; *p* < 0.05 was considered statistically significant. TMTH: *Tenebrio molitor* larva trypsin hydrolysate, MNX: minoxidil.

To further investigate the molecular mechanisms underlying hair growth promotion, the expression of key proteins involved in the Wnt/β-catenin and PI3K/Akt signaling pathways, including Wnt3a, β-catenin, PI3K, Akt, phosphorylated Akt, GSK3β, phosphorylated GSK3β, and androgen receptor (AR), was analyzed in the dorsal skin of TP-induced androgenetic alopecia mice. In the TP group, the expression levels of Wnt3a, β-catenin, PI3K, phosphorylated Akt, and phosphorylated GSK3β were reduced compared with those in the control group, whereas AR expression was increased. In contrast, TMTH treatment restored the expression of Wnt/β-catenin- and PI3K/Akt-related proteins and showed a decreasing trend in AR expression. Similar changes were observed in the minoxidil-treated positive control group. These results suggest that TMTH may restore hair growth-related signaling pathways, including the Wnt3a/β-catenin and PI3K/Akt/GSK3β axes, while modulating local AR-associated responses in dorsal skin tissue (Fig. 6).

### TMTH Promotes Anti-Oxidative Signaling and Suppresses TNF-α Expression in the Dorsal Skin of Testosterone Propionate-Induced C57BL/6 Mice

**Fig. 7:**
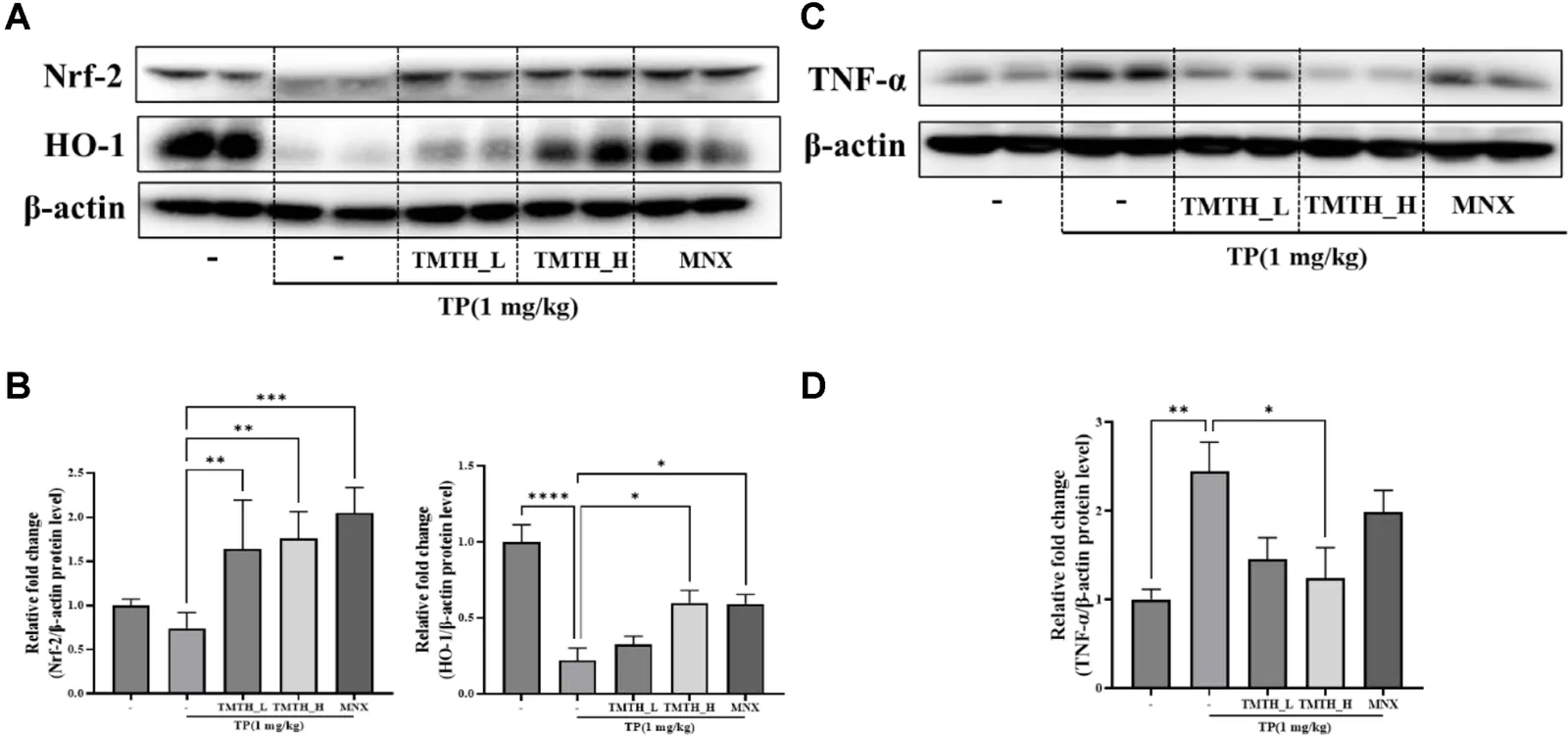
Effects of TMTH on anti-oxidative signaling and TNF-α expression in dorsal skin of TP-induced mice. (**A**) Representative western blot images for Nrf2, HO-1, and β-actin. (**B**) Quantitative analysis of Nrf2/β-actin and HO-1/β-actin protein levels. (**C**) Representative western blot images for TNF-α and β-actin. (**D**) Quantitative analysis of TNF-α/β-actin protein levels. β-actin served as a loading control. Data are means ± SEM from three independent experiments. Statistical analysis was performed using one-way ANOVA followed by Dunnett's test; *p* < 0.05. TMTH: *Tenebrio molitor* larva trypsin hydrolysate, MNX: minoxidil.

TP-induced AR upregulation may be associated with increased inflammation and oxidative stress in the dorsal skin. Western blot analysis revealed that the expression of Nrf2 and HO-1, proteins that suppress oxidative stress, was reduced in the TP group while being elevated in the TMTH and minoxidil-treated groups. Conversely, the expression of TNF-α was increased in the TP group but reduced by TMTH treatment. These results suggest that TMTH may create a favorable environment for hair growth by enhancing antioxidant-related signaling and reducing inflammatory responses (Fig. 7).

## Discussion

Alopecia is a critical medical issue that can have significant negative psychological and aesthetic impacts on individuals, highlighting the need to develop effective and safe treatments for alopecia [24-25]. Among the various types of alopecia, AGA is the most common and affects approximately 80% of men and more than 50% of women with age [26]. AGA is characterized by progressive hair thinning and loss caused by shortening of the anagen phase and prolongation of the telogen phase of the hair cycle [13]. The hair cycle is regulated in part by the action of androgens on hair dermal papilla cells. During the anagen phase, activation of the Wnt/β-catenin signaling pathway promotes hair growth by inducing the production of growth factors. However, testosterone can be converted into DHT, a more potent androgen, by 5α-reductase. DHT binds strongly to androgen receptors and can interfere with Wnt/β-catenin signaling, thereby shortening the anagen phase and prolonging the telogen phase [27-28]. Recent studies have also demonstrated that elevated oxidative stress and inflammation within hair follicles inhibit hair growth and contribute to the progression of AGA [29-30].

Currently available treatments for AGA, including finasteride and minoxidil, have limitations. Finasteride reduces DHT production by inhibiting 5α-reductase, but it may be associated with adverse effects related to sexual function. Minoxidil is widely used as a hair growth-promoting agent, but local adverse effects, such as scalp irritation and itching, may occur in susceptible individuals [11-13]. Therefore, additional functional ingredients with improved tolerability and multi-target biological activity are worth investigating.

Natural compounds derived from protein hydrolysis have recently gained recognition as biological regulators due to their ability to exert physiological functions in the body. This makes them promising candidates for incorporation into functional foods aimed at disease prevention and health management. Numerous studies have documented the enzymatic hydrolysis of insect proteins to produce bioactive peptides, emphasizing their potential as functional ingredients. The bioactivity and functionality of these peptides are determined by their amino acid composition and length, which are influenced by the choice of enzyme and the conditions under which hydrolysis occurs [31]. *T. molitor* is considered a promising sustainable protein source, and recent research has reported a range of benefits of *T. molitor*, including anti-diabetic, anti-hypertensive, anti-inflammatory, and antioxidant properties [21]. In the present study, various *T. molitor* larva hydrolysates were screened, and TRY showed the strongest effect on HDP cell proliferation and β-catenin expression (Fig. 1). Therefore, TRY was selected and designated as *Tenebrio molitor* larva trypsin hydrolysate (TMTH) for subsequent experiments.

The Wnt/β-catenin signaling pathway regulates several processes, such as skin wound remodeling, hair follicle morphogenesis, hair shaft growth, and the hair cycle. β-Catenin is particularly crucial for epithelial cell differentiation into hair follicles that are responsible for producing the hair shaft and inner root sheath. Additionally, it facilitates the transition from the telogen to the anagen phase [32]. In the present study, immunofluorescence staining confirmed increased β-catenin expression in HDP cells treated with TMTH (Fig. 2). In DHT-induced HDP cells, TMTH restored cell proliferation and migration suppressed by DHT (Fig. 3). These findings suggest that TMTH may improve AGA-related cellular dysfunction by modulating β-catenin expression and promoting HDP cell proliferation and migration.

In HDP cells, glycogen synthase kinase-3β (GSK3β) inhibits β-catenin, thereby suppressing its activity and inhibiting hair growth [33]. Additionally, the AR activates GSK3β, further suppressing β-catenin activity [34]. Therefore, inhibition of both AR and GSK3β may promote hair growth.

In a mouse model with TP-induced hair loss, TMTH increased the hair-covered area, follicle number, and hair shaft diameter (Figs. 4 and 5). Additionally, TMTH enhanced the expression of key proteins involved in the Wnt/β-catenin and PI3K/Akt signaling pathways, including Wnt3a, β-catenin, PI3K, phosphorylated Akt, and phosphorylated GSK3β, while showing a decreasing trend in AR expression (Fig. 6). These findings suggest that TMTH may restore hair growth-related signaling pathways suppressed by TP-induced androgenic stress. In particular, activation of PI3K/Akt signaling can promote the phosphorylation and inactivation of GSK3β, thereby reducing β-catenin degradation and facilitating its accumulation. Increased Wnt3a expression may further contribute to β-catenin-dependent signaling. Consequently, the stabilization and activation of β-catenin may promote the transition of hair follicles into the anagen phase and stimulate hair growth [35]. Therefore, TMTH may exert its hair growth-promoting effects by modulating local AR-associated responses and activating the Wnt3a/β-catenin and PI3K/Akt/GSK3β signaling pathways.

Although minoxidil is generally known as an ATP-sensitive potassium channel opener, previous studies have reported that minoxidil may suppress AR-related functions, including AR transcriptional activity and AR target protein expression [36]. Therefore, the reduction of AR expression observed in the minoxidil-treated group may reflect improvement of the TP-induced alopecic microenvironment and modulation of local androgen-related responses rather than systemic anti-androgenic activity. From an applied food-material perspective, edible insects are also recognized for their nutritional and sensory quality, which further supports their consideration as sustainable food-derived functional ingredients [37].

TMTH also exhibited antioxidant and anti-inflammatory effects. It increased the expression of antioxidant proteins Nrf2 and HO-1 while reducing the expression of the inflammatory cytokine TNF-α, creating a favorable environment for hair growth (Fig. 7). In contrast, minoxidil did not reduce TNF-α expression under the present experimental conditions. This observation requires further investigation but may be relevant to local irritation or inflammatory responses reported in some cases of minoxidil use. Furthermore, the anti-inflammatory effects of TMTH have been corroborated in recent studies, further supporting the findings of the current research [18].

Although TMTH showed hair growth-promoting effects in HDP cells and in the TP-induced alopecia mouse model, several limitations should be addressed in future studies. First, TMTH was used as a whole trypsin-mediated enzymatic hydrolysate in the present study, and the biological activity of the crude hydrolysate was evaluated. Therefore, further chemical characterization, including molecular weight distribution analysis, degree of hydrolysis measurement, peptide fractionation, purification of active peptides, and LC-MS/MS-based peptide profiling, would be useful to improve reproducibility and support the future development of TMTH as a functional ingredient. Second, minoxidil was used as a positive control because it is widely used as a reference drug in androgenetic alopecia and hair growth studies. Although finasteride is also an important reference drug for AGA, comparison with finasteride would require additional androgen metabolism-related analyses, including 5α-reductase activity, DHT levels, systemic androgen levels, and androgen-related safety assessments. Third, although TMTH reduced TP-induced AR expression in dorsal skin tissue, this result should be interpreted carefully as modulation of local androgen-related responses in the hair follicle microenvironment rather than direct evidence of systemic androgen suppression. Since serum testosterone levels, DHT levels, reproductive organ weight, and sexual-function-related safety parameters were not evaluated in the present study, further studies are required to determine whether TMTH affects androgen metabolism or has finasteride-like effects. Taken together, TMTH may serve as a potential functional ingredient candidate for androgenetic alopecia by restoring DHT- or TP-suppressed hair growth-related responses and by modulating Wnt/β-catenin, PI3K/Akt, antioxidant, and inflammatory pathways.

## Supplemental Materials

Supplementary data for this paper are available on-line only at http://jmb.or.kr.



## References

[1] Hunt N, McHale S (2005). The psychological impact of alopecia. BMJ.

[2] Qi J, Garza LA (2014). An overview of alopecias. Cold Spring Harb. Perspect. Med..

[3] Devjani S, Ezemma O, Kelley KJ, Stratton E, Senna M (2023). Androgenetic alopecia: Therapy update. Drugs.

[4] Rathnayake D, Sinclair R (2010). Male androgenetic alopecia. Expert Opin. Pharmacother..

[5] Schneider MR, Schmidt-Ullrich R, Paus R (2009). The hair follicle as a dynamic miniorgan. Curr. Biol..

[6] Park S-M, He Y-C, Gong C, Gao W, Bae Y-S, Si C (2022). Effects of taxifolin from enzymatic hydrolysis of Rhododendron mucrotulatum on hair growth promotion. Front. Bioeng. Biotechnol..

[7] Choi BY (2020). Targeting Wnt/β-catenin pathway for developing therapies for hair loss. Int. J. Mol. Sci..

[8] Liu D, Xu Q, Meng X, Liu X, Liu J (2024). Status of research on the development and regeneration of hair follicles. Int. J. Med. Sci..

[9] Trüeb RM (2002). Molecular mechanisms of androgenetic alopecia. Exp. Gerontol..

[10] Trüeb RM, Dias MFRG (2018). Alopecia areata: a comprehensive review of pathogenesis and management. Clin. Rev. Allergy Immunol..

[11] Tang Y, Wang C, Desamero MJM, Kok MK, Chambers JK, Uchida K (2023). The Philippines stingless bee propolis promotes hair growth through activation of Wnt/β-catenin signaling pathway. Exp. Anim..

[12] Lee SW, Juhasz M, Mobasher P, Ekelem C, Mesinkovska NA (2018). A systematic review of topical finasteride in the treatment of androgenetic alopecia in men and women. J. Drugs Dermatol..

[13] Nestor MS, Ablon G, Gade A, Han H, Fischer DL (2021). Treatment options for androgenetic alopecia: efficacy, side effects, compliance, financial considerations, and ethics. J. Cosmet. Dermatol..

[14] Kotsou K, Chatzimitakos T, Athanasiadis V, Bozinou E, Athanassiou CG, Lalas SI (2023). Innovative applications of *Tenebrio molitor* larvae in food product development: a comprehensive review. Foods.

[15] Rumpold BA, Schlüter OK (2013). Nutritional composition and safety aspects of edible insects. Mol. Nutr. Food Res..

[16] Nowakowski AC, Miller AC, Miller ME, Xiao H, Wu X (2022). Potential health benefits of edible insects. Crit. Rev. Food Sci. Nutr..

[17] Song Y, Gu H, Jo JM, Shin M, Kim SY, Gam DH (2020). Production of functional peptide with anti-obesity effect from defatted *Tenebrio molitor* larvae using proteolytic enzyme. Biotechnol. Bioprocess Eng..

[18] Fan M, Wedamulla NE, Choi YJ, Zhang Q, Bae SM, Kim EK (2023). *Tenebrio molitor* larva trypsin hydrolysate ameliorates atopic dermatitis in C57BL/6 mice by targeting the TLR-mediated MyD88-dependent MAPK signaling pathway. Nutrients.

[19] Kim SW, WG Sang, WJ Chi, WY Bang, SJ Park, TW Kim, *et al*. 2023. Antioxidant properties of peptides extracted from Tenebrio molitor larvae. *J. Life Sci.* **33:** 383-390. https://doi.org/10.5352/JLS.2023.33.5.383. 10.5352/JLS.2023.33.5.383

[20] Brai A, Trivisani CI, Vagaggini C, Stella R, Angeletti R, Iovenitti G (2022). Proteins from *Tenebrio molitor*: an interesting functional ingredient and a source of ACE inhibitory peptides. Food Chem..

[21] Wedamulla NE, Zhang Q, Kim S, Choi Y, Bae SM, Kim E (2024). Korean edible insects: a promising sustainable resource of proteins and peptides for formulating future functional foods. Food Suppl. Biomater. Health.

[22] Zhu N, Yan J, Gu W, Yang Q, Lin E, Lu S (2024). Dermal papilla cell-secreted biglycan regulates hair follicle phase transit and regeneration by activating Wnt/β-catenin. Exp. Dermatol..

[23] Wang Z, Feng Y, Ma L, Li X, Ding W, Chen X (2017). Hair growth promoting effect of white wax and policosanol from white wax on the mouse model of testosterone-induced hair loss. Biomed. Pharmacother..

[24] Gordon KA, Tosti A (2011). Alopecia: evaluation and treatment. Clin. Cosmet. Investig. Dermatol..

[25] Moattari CR, Jafferany M (2022). Psychological aspects of hair disorders: consideration for dermatologists, cosmetologists, aesthetic, and plastic surgeons. Skin Appendage Disord..

[26] Kaiser M, Abdin R, Gaumond SI, Issa NT, Jimenez JJ (2023). Treatment of androgenetic alopecia: current guidance and unmet needs. Clin. Cosmet. Investig. Dermatol..

[27] Abdin R, Zhang Y, Jimenez JJ (2022). Treatment of androgenetic alopecia using PRP to target dysregulated mechanisms and pathways. Front. Med..

[28] Kidangazhiathmana A, Santhosh P (2022). Pathogenesis of androgenetic alopecia. Clin. Dermatol. Rev..

[29] Du F, Li J, Zhang S, Zeng X, Nie J, Li Z (2024). Oxidative stress in hair follicle development and hair growth: signalling pathways, intervening mechanisms and potential of natural antioxidants. J. Cell. Mol. Med..

[30] Oiwoh SO, Enitan AO, Adegbosin OT, Akinboro AO, Onayemi EO (2024). Androgenetic alopecia: a review. Niger. Postgrad. Med. J..

[31] Rivero-Pino F, Pérez Gálvez R, Espejo Carpio FJ, Guadix EM (2020). Evaluation of *Tenebrio molitor* protein as a source of peptides for modulating physiological processes. Food Funct..

[32] Fu H, Li W, Weng Z, Huang Z, Liu J, Mao Q (2023). Water extract of cacumen platycladi promotes hair growth through the Akt/GSK3β/β-catenin signaling pathway. Front. Pharmacol..

[33] Piao S, Lee S-H, Kim H, Yum S, Stamos JL, Xu Y (2008). Direct inhibition of GSK3β by the phosphorylated cytoplasmic domain of LRP6 in Wnt/β-catenin signaling. PLoS One.

[34] Hong G, Lee H, Kim Y, Kim K, Jung J (2023). *Stauntonia hexaphylla* extract ameliorates androgenic alopecia by inhibiting androgen signaling in testosterone-induced alopecia mice. Iran. J. Pharm. Res..

[35] Kwack MH, Kang BM, Kim MK, Kim JC, Sung YK (2011). Minoxidil activates β-catenin pathway in human dermal papilla cells: a possible explanation for its anagen prolongation effect. J. Dermatol. Sci..

[36] Hsu CL, Liu JS, Lin AC, Yang CH, Chung WH, Wu WG (2014). Minoxidil may suppress androgen receptor-related functions. Oncotarget.

[37] Kouřimská L, Adámková A (2016). Nutritional and sensory quality of edible insects. NFS J..

