# Supplementary material for: Inhibitory Effects of Tenebrio molitor Larva Trypsin Hydrolysate on Androgenetic Alopecia
Source: J Microbiol Biotechnol. 2026 Aug 14;36:e2606002. doi: 10.4014/jmb.2606.06002 (PMC13591393; doi:10.4014/jmb.2606.06002)

Supplementary Data 1

J. Microbiol. Biotechnol. 2026. 36: e2606002
https://doi.org/10.4014/jmb.2606.06002

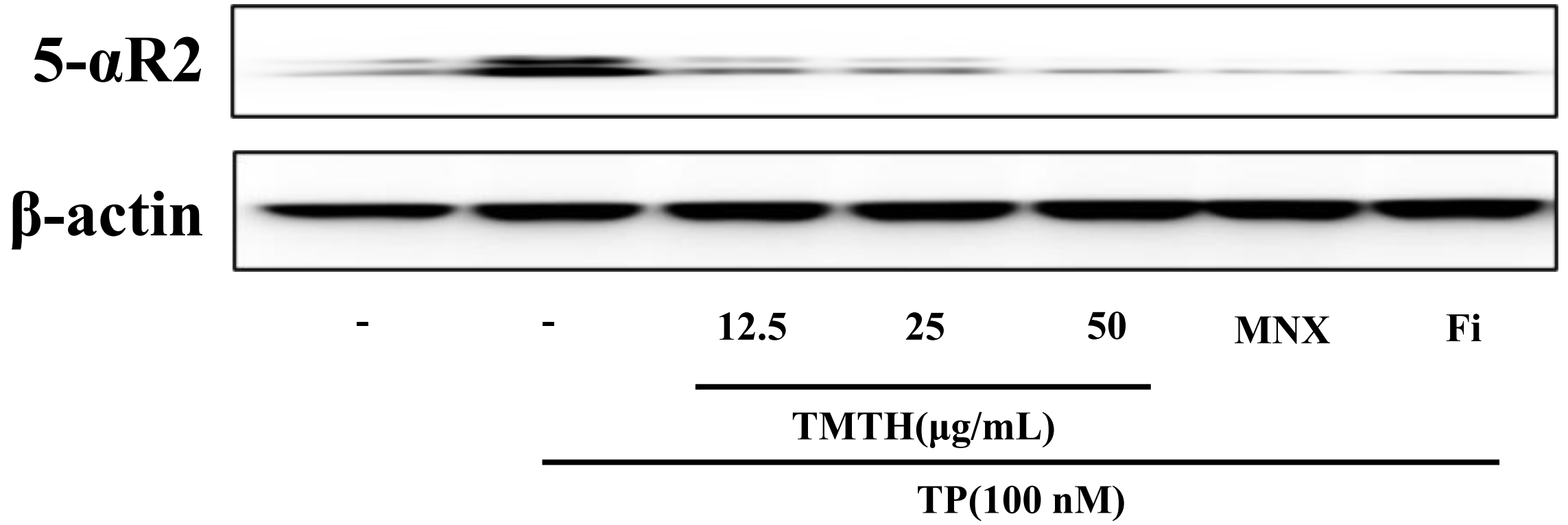

Supplementary Data 2

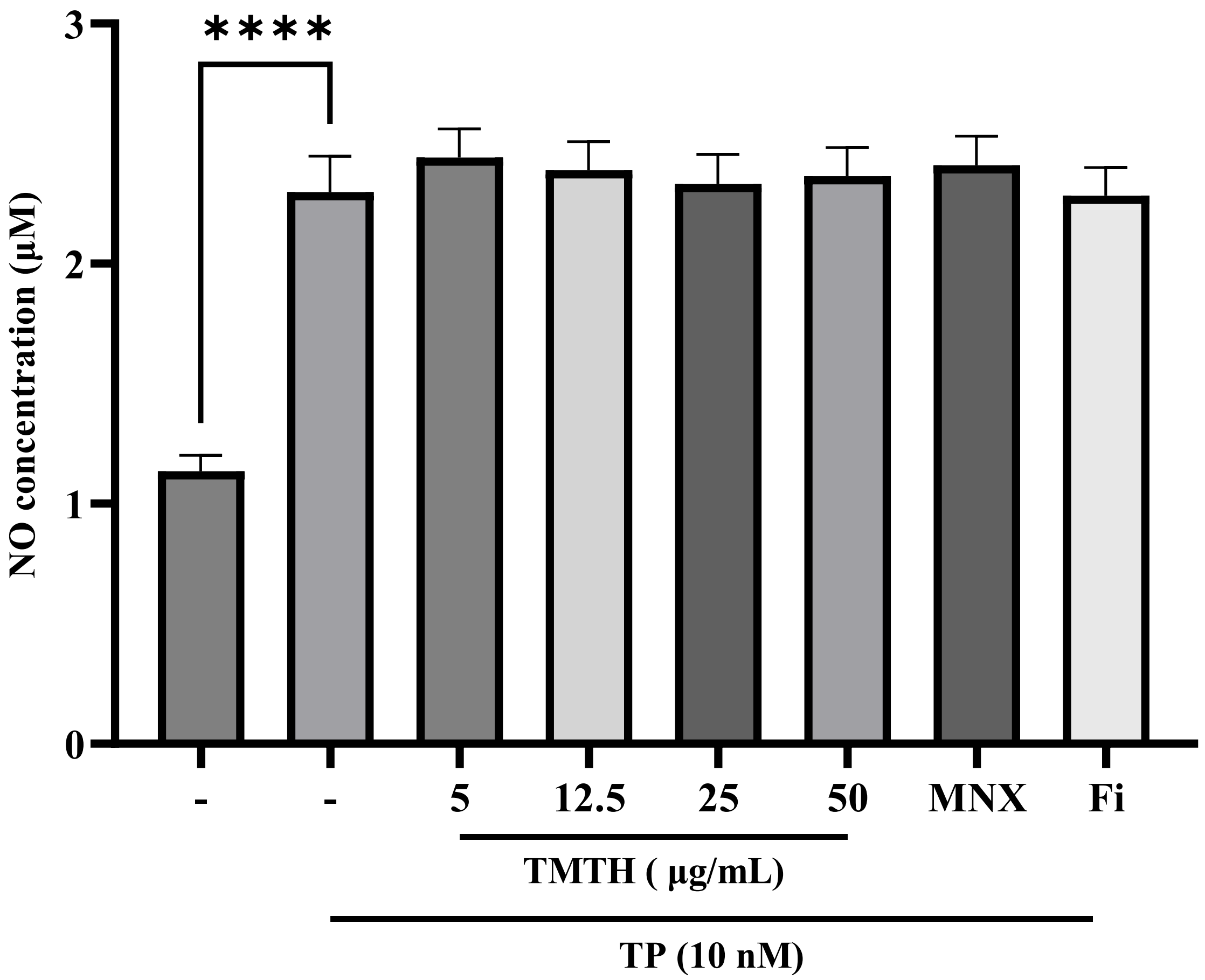

- Western blot_ fig 1.

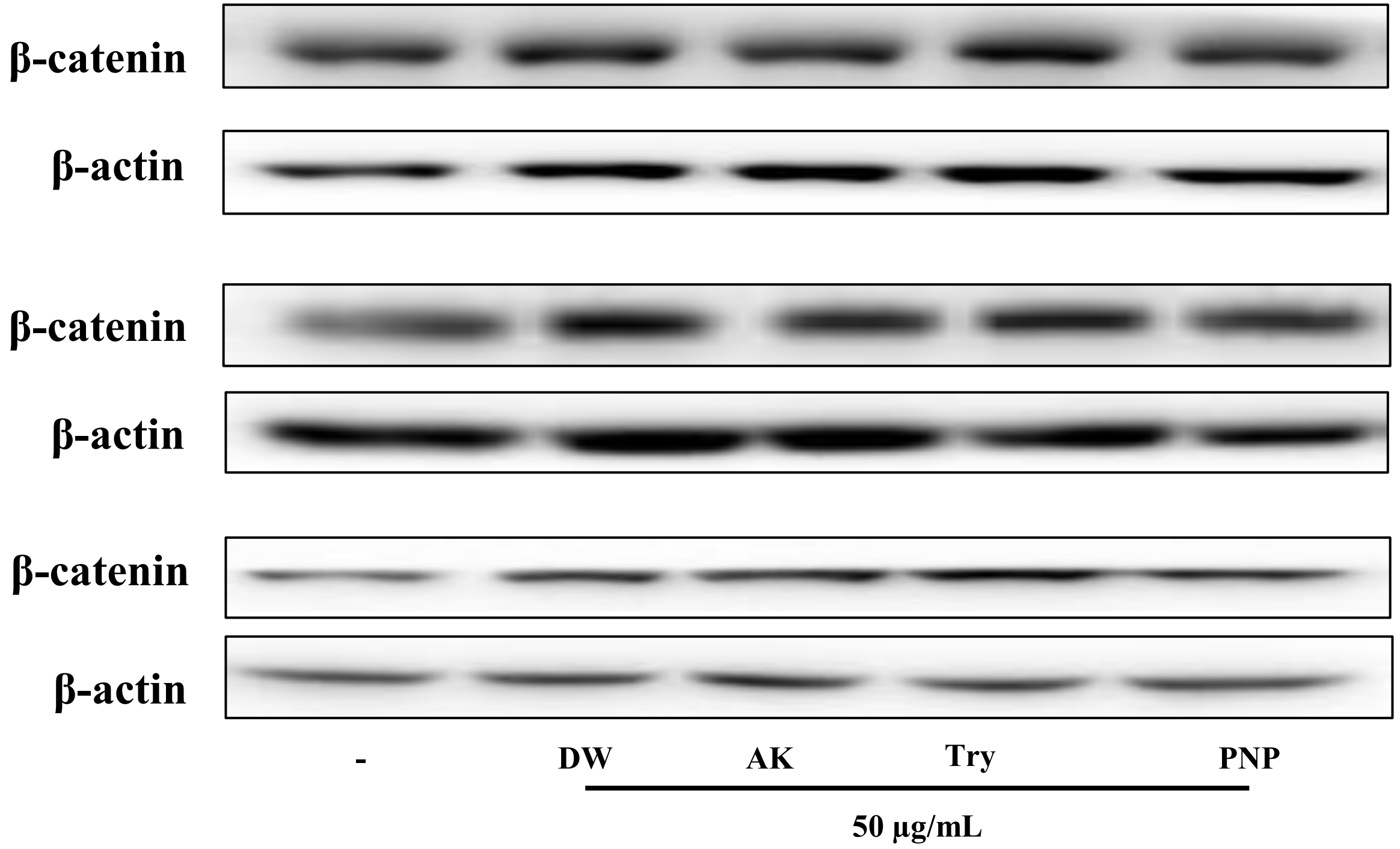

Supplement: Supplementary file 1 [file jmb-36-e2606002-supple.pdf]
